# Fluorescence Enhanced Optical Resonator Constituted of Quantum Dots and Thin Film Resonant Cavity for High-Efficiency Reflective Color Filter

**DOI:** 10.3390/nano11112813

**Published:** 2021-10-23

**Authors:** Xiaochuan Chen, Pengxia Liang, Qian Wu, Qiaofeng Tan, Xue Dong

**Affiliations:** 1 State Key Laboratory of Precision Measurement Technology and Instruments, Department of Precision Instrument, Tsinghua University, Beijing 100084, China; chenxiaochuan@boe.com.cn; 2 BOE Technology Group Co., Ltd., Beijing 100176, China; liangpengxia@boe.com.cn (P.L.); wuqian-cto@boe.com.cn (Q.W.)

**Keywords:** color filter, quantum dots, fluorescence enhanced optical resonator

## Abstract

Conventional color filters selectively absorb a part of the backlight while reflecting or transmitting other light, resulting in the problem of low efficiency and energy wasting. For this problem, a new concept of fluorescence enhanced optical resonator was proposed and verified in this paper. The new structure consists of structural color filter and light-conversion material. Specially, a thin film resonant cavity was designed, and InP/ZnSe/ZnS quantum dots were inserted inside the resonator. When illuminated by sunlight, the novel fluorescence enhanced optical resonator could not only reflect the specific light, but also convert absorbed energy into desired light, leading to the utilization efficiency improvement of solar energy. An all-dielectric red fluorescence enhanced optical resonator was fabricated, with peak equivalent reflectance up to 105%. Compared with a thin film resonator, the enhancement coefficient of the as-proposed structure is about 124%. The new optical structure can utilize solar source efficiently, showing application potential as the next generation of reflective color filters for display.

## 1. Introduction

Demands for panel display devices, which is one of the most important ways to communicate and share information, are dramatically increasing, especially during the COVID-19 pandemic. With the advantages of low energy consumption, good behavior in bright light, and good eye-care characteristics, reflective display technology has attracted tremendous attention in recent years [1,2,3,4]. However, most commercial reflective display products can only exhibit black and white pictures. One of the reasons is the introduction of color filer will loss 70% of light energy, which will weaken the advantage of low energy consumption and even cause failure to display.

The color filter, which is commonly utilized as a wave band-selective filter in the visible region, can be traditionally divided into pigment color filters and structural color filters [5,6,7]. The pigment color filter realizes wave band selection by absorbing light with a specific wavelength, while the structural color filter does so through the interference principle of specific geometric structures, such as multilayer film or photonic crystal. Lots of structural color filter based on Fabry–Perot (F–P) cavity [8,9] and guided mode resonant grating filters [10,11] have also been studies. No matter the type of conventional color filters, the basic principle of absorbing undesired wavelength light results in the low effective utilization of the source energy.

Quantum dots are one kind of zero-dimensional materials. With the special spatial structure, quantum dots demonstrate lots of distinctive optical properties, which are utilized in sensing [12,13], diagnosis [14,15] and imaging [16,17]. One of the characteristics of quantum dots is that they can be used as light conversion materials [18,19]. Chen et al. utilized this kind of quantum dot to develop thin films with a negative extinction coefficient and made extra high reflection mirrors [20]. Basically, quantum dots can absorb light energy by band gap transition and then radiate specific wavelength light depending on the width of the energy band. Particularly, the wavelength of luminescence light can be changed by varying the size of the quantum dots [21]; the full width of the half maximum is usually thinner than traditional pigment, which means that the quantum dots can achieve a larger color gamut [22]. With the above advantages, quantum dots are widely used in display and considered the next generation of color filters.

**Figure 1:**
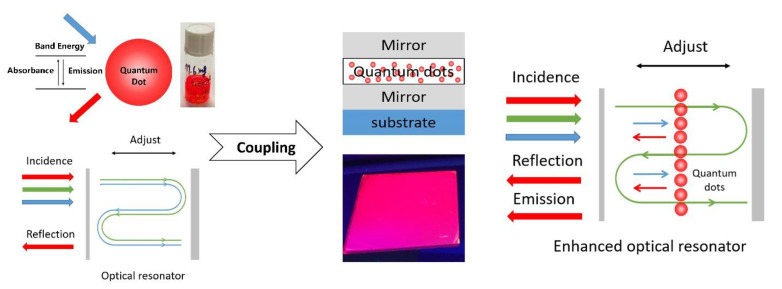
Scheme of the Fluorescence enhanced optical resonator.

In this paper, a new concept of fluorescence enhanced optical resonator, which couples an optical resonator and fluorescent quantum dots, is proposed for the first time. Figure 1 shows the schematic diagram of the proposed resonator. Inside an optical resonator with specific cavity length, a thin film of quantum dots was prepared, making up the fluorescence enhanced optical resonator, and all the parameters matched perfectly. The as-proposed optical structure is supposed to not only reflect the desired part of incident light, but also “produce” desired light via the property of quantum dots, which can improve efficiency for solar energy utilization. With the characteristics mentioned, the fluorescence enhanced optical resonator has application potentials for high-efficiency reflective color filters.

## 2. Materials and Methods

### 2.1. Design Principles of Fluorescence Enhanced Optical Resonator

**Figure 2:**
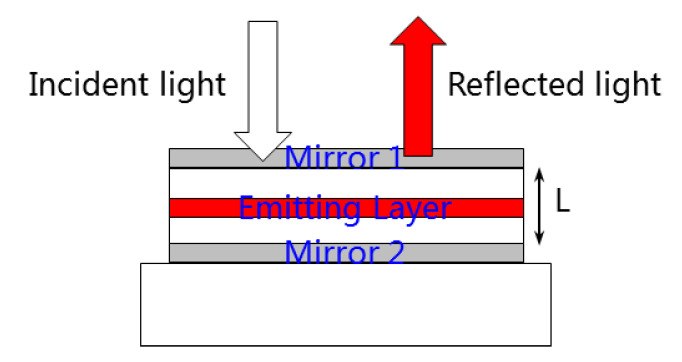
Schematic diagram of fluorescence enhanced optical resonator.

Figure 2 shows the schematic diagram of the as-designed fluorescence enhanced optical resonator. The detectable outgoing light is composed of two parts: (1) the reflected portion when the incident light is projected onto the resonant cavity; and (2) the fluorescence generated by the stimulated quantum dots when the transmitting portion in the incident light enters into the resonant cavity. Basically, the enhancement cavity is an optical structure, consisting of a total reflection mirror, a partial reflection mirror and a light conversion layer between them. The two mirror layers are designed to form a thin film resonator, while the light conversion layer is used to improve the efficiency of source utilization.

Take the red light enhanced type as an example. The design of enhanced resonator preferentially ensures the reflectivity of desired red light. Additionally, the short-wavelength light should be insured to enter the fluorescent layer of the resonant cavity as much as possible. The fluorescent layer, such as quantum dots, absorbs the short-wavelength light and converts energy into red light, which is used to enhance the overall red-light reflectivity of the device. The enhanced resonator is capable of spectrally modulating the illuminant placed inside and achieving radiation enhancement at the resonant mode. This is because the cavity, which is comparable in length to the optical wavelength, greatly changes the mode density within the cavity so that the electromagnetic field mode within the cavity, instead of being continuously distributed as in free space, presents a discrete distribution. In addition, the rate of spontaneous emission of the radiation dipole placed in the cavity is determined not only by the mode density within the cavity, but also by the strength of the coupling between the excited dipole and the constrained electromagnetic field. When the radiation dipole is in different positions of the cavity, its spontaneous emission rate will also be different. This is because the standing wave electric field in the planar micro-cavity is distributed as a sinusoidal function with different intensity values at different positions. It has a maximum at the antinode and a minimum at the node. According to the above reasons, the enhanced resonator should also make the quantum dots at the antinode of the electric field in the micro-cavity effect through reasonable film design so as to maximize the electromagnetic coupling of quantum dots and incoming short-wavelength light and improve the effective utilization rate of the quantum dots.

Taking the reflection peak at 620 nm and the quantum dot absorption peak at 450 nm as an example, the equivalent reflectivity of the enhanced resonator can be calculated by the following formula:

(1) $$R_{620}=R_{620}{}^{\prime }+\frac{{\left|E_{cavity}\right|}^{2}}{{\left|E_{620}\right|}^{2}}=R_{620}{}^{\prime }+\frac{{\left|E_{\mathrm{CC}}\right|}^{2}\times G_{cavity}}{{\left|E_{620}\right|}^{2}}$$

where $R_{620}$ represents the reflectance of the enhanced resonator at 620 nm, $R_{620}{}^{\prime }$ represents the reflectance of the normal resonator at 620 nm, $\left|E_{\mathrm{CC}}\right|$ represents the amplitude of 620 nm light obtained by quantum dots conversion, $\left|E_{620}\right|$ represents the amplitude of 620 nm from incident light, and $G_{cavity}$ represents the coefficient between the light emitting from the outer boundary and the actually converted light. It can be seen that the fluorescence emission spectrum of the enhanced resonator is obtained by multiplying the intrinsic spectrum of the fluorescent material (usually its PL (Photoluminicence) spectrum) and the micro-cavity resonance spectrum.

(2) $${\left|E_{\mathrm{CC}}\right|}^{2}=N_{620}h\frac{c}{\lambda_{620}}\approx {\left|E_{450}\right|}^{2}\times \alpha_{\mathrm{absorb}}\times PLQY\times \frac{\lambda_{450}}{\lambda_{620}}$$

This formula calculates the intrinsic energy conversion ability of fluorescent material, where $\alpha_{\mathrm{absorb}}$ represents the absorptance, and PLQY represents the photoluminescence quantum efficiency of the material [23].

(3) $$G_{cavity}\left(\lambda \right)=\frac{\left(1-R_{2})[1+R_{1}+2\sqrt{R_{1}}\cos(4\pi z_{\mathrm{eff}}/\lambda +\varphi_{2})\right]}{1+R_{1}R_{2}-2\sqrt{R_{1}R_{2}}\cos(4\pi L_{\mathrm{eff}}/\lambda +\varphi_{1}+\varphi_{2})}$$

The micro-cavity coefficient can be calculated based on formula (3) [24], where *R*_1_ and *R*_2_ are the reflectivity corresponding to the upper and lower mirrors of the device respectively, z_eff_ represents the equivalent optical length between the light-emitting exciton and the lower mirror M_2_, *L*_eff_ represents the equivalent optical length of the whole micro-cavity device, and *φ*_1_ and *φ*_2_ are the reflection phase changes of M_1_ and M_2_ metal mirrors, respectively.

Since Leff is defined by the characteristics of the reflective resonator, as shown in Figure S1, in order to obtain the maximum output light intensity of the enhanced resonator, it is required to meet the demands of the following formula:

(4) $$\frac{4\pi z_{\mathrm{eff}}}{\lambda }+\varphi_{1}=2k\pi (k=0,1,2,\ldots )$$

It can be proved that this position is the antinode of the electric field in the micro-cavity at which the optical conversion of quantum dots can be stimulated most effectively.

### 2.2. Preparation of Fluorescence Enhanced Optical Resonator

**Figure 3:**
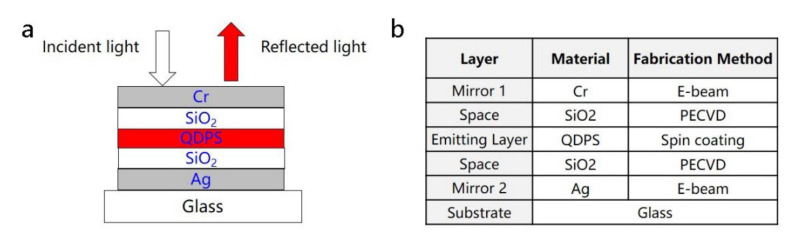
(**a**) Basic structure of fluorescence enhanced optical resonator; (**b**) fabrication method for different layers.

Based on the design principles above, one basic structure of the fluorescence enhanced optical resonator and the fabrication method are shown in Figure 3. All the thin films were prepared on a substrate glass. Silver (Ag) was chosen as the material of Mirror 2 because of the high reflection. An electronic beam (E-beam) was utilized to heat the Ag source, which went from a condensed phase to a vapor phase. With being in contact with the substrate glass, Ag then went back to a thin film-condensed phase. This method can form a uniform thin film in nano-scale. The material of space layer was silicon oxide (SiO_2_), which was prepared by plasma enhanced chemical vapor deposition (PECVD). The space layer could also be titanium oxide (TiO_2_), prepared by atomic layer deposition (ALD). SiO_2_ and TiO_2_ have different refractive indexes, which can be utilized to adjust the cavity structure to obtain high reflectance for a specific wavelength spectrum. The most important layer in the fluorescence enhanced optical resonator is quantum dot-polystyrene layer (QDPS). InP/ZnSe/ZnS core-shell quantum dots were utilized here. Both InP/ZnSe/ZnS quantum dots and polystyrene were dispersed into toluene, and then the mixture was spin-coated to form the nano-scale thin film. Mirror 1 was also prepared by the E-beam method. Differently, Cr would be a better choice with its property of n≈k, according to reference [8]. The thickness of all the material layers can be monitored by the quartz microbalance in the instruments and checked by transmission electron microscope (TEM). The final fluorescence enhanced optical resonator would be more complex. Commercial software FDTD, TFCalc and Macleod, were used to optimize the thickness and material of different layers.

## 3. Results and Discussion

### 3.1. Characterization of Quantum Dots

**Figure 4:**
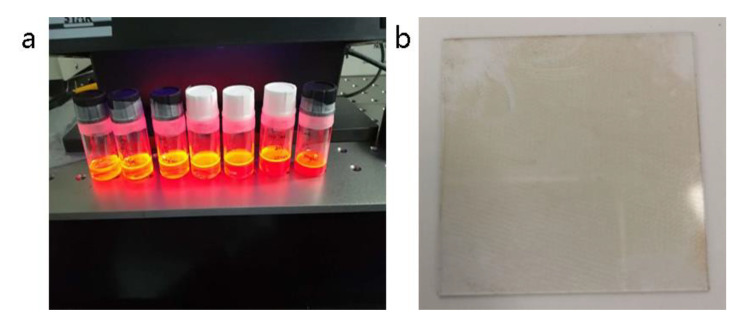
(**a**) InP/ZnSe/ZnS quantum dots and polystyrene dispersed in toluene; (**b**) QDPS thin film prepared by spin coating method.

Compared with conventional resonator devices, the most significant difference of fluorescence enhanced optical resonator in this paper is the introduction of quantum dots with light conversion function. InP/ZnSe/ZnS core-shell quantum dots, with a quantum yield as high as 80%, were utilized. As depicted in Figure 4a, InP/ZnSe/ZnS quantum dots and polystyrene were dispersed into toluene, and the appropriate quantum dot concentration ratio could be achieved by adjusting the dose of the solvent. The function of polystyrene was to form the quantum dots thin film with a controllable thickness and avoid aggregation quenching. Afterwards, the mixture solution was transferred to a substrate glass by the spin coating method, and the thickness could be adjusted with rotating speed. Figure 4b shows the QDPS thin film on the glass.

**Figure 5:**
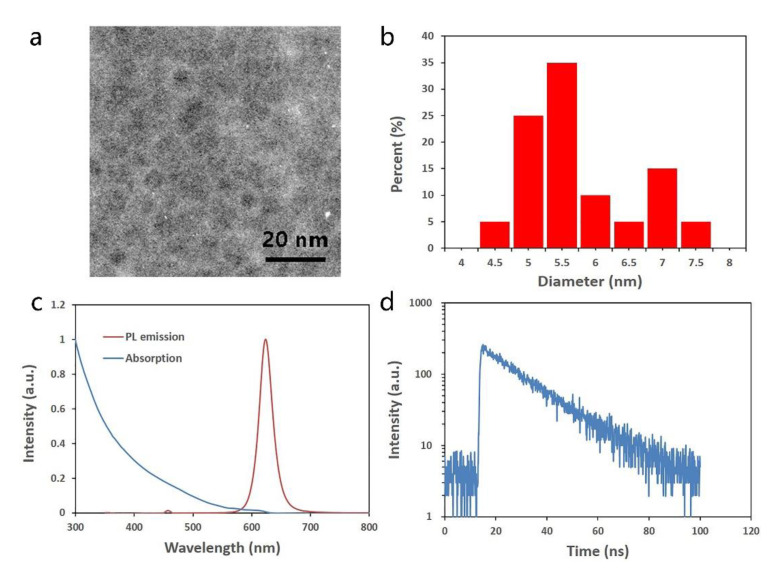
Characterization of as-used InP/ZnSe/ZnS quantum dots: (**a**) TME image; (**b**) particle size distribution histogram; (**c**) UV-visual absorption and photoluminescence emission spectra; (**d**) fluorescence lifetime.

To better understand the properties of fluorescence enhanced optical resonator, InP/ZnSe/ZnS were completely characterized, as shown in Figure 5. Figure 5a shows the morphology of the as-used quantum dots, which are monodisperse nanoparticles. Based on the TEM image, the size of the quantum dots has to be statistically analyzed. Utilizing distribution histogram and the Gauss equation fitting method, the average diameter was counted to be 5.79 nm, with a relatively low volatility. The optical properties were further characterized. As shown in Figure 5c, the absorption of InP/ZnSe/ZnS quantum dots decreases with the increase in wavelength ranging from 300 nm to 800 nm. The result shows that the blue light will excite the quantum dots effectively, just as expected. The peak of the photoluminescence emission spectrum, stimulated by 400 nm light, is at 625 nm, with full width at half maximum (FWHM) of ~30 nm. The small FWHM demonstrates the advantage of quantum dots in a high color purity display field. Interestingly, a relatively small emission peak can be found at ~460 nm. Considering the core-shell structure of InP/ZnSe/ZnS, this large photoluminescence peak is due to core InP, while this small one may result from shell ZnSe/ZnS. The different bandgap valves of InP and ZnSe/ZnS may lead to this phenomenon, according to reference [25]. Figure 5d depicts the fluorescence lifetime of quantum dots, which was measured to be 16.7 ns. The longer lifetime means fewer defects, which is the benefit of the core-shell structure.

### 3.2. Metal–Dielectric Fluorescence Enhanced Optical Resonator

**Figure 6:**
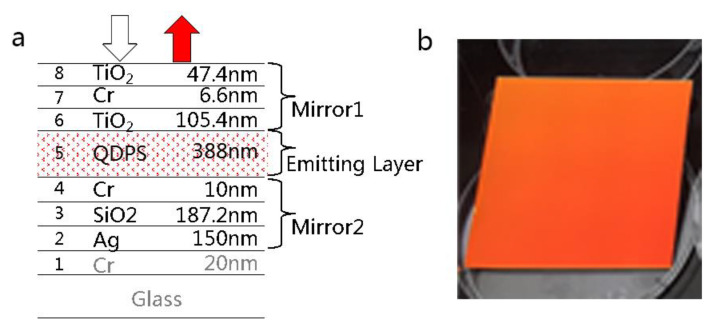
(**a**) Structure of metal–dielectric fluorescence enhanced optical resonator and (**b**) the picture of as-designed sample.

The metal–dielectric fluorescence enhanced optical resonator is composed of two mirrors and fluorescent material inside. The specific film information is shown in Figure 6a, in which Mirror 1 is made of Cr, while Mirror 2 is made of Ag. Additionally, in order to suppress the reflection of blue light, a layer of Cr is added at the side of Mirror 2. This layer can effectively improve the color purity of reflected light. Figure 6b shows the picture of the as-designed sample, which appears red under the irradiation of natural light. The structure meets the demand for color purity of the reflected color filter.

**Figure 7:**
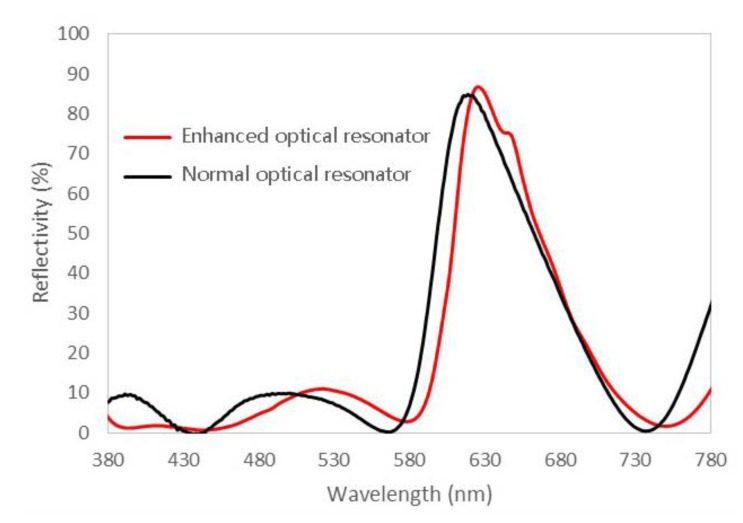
Measured reflection spectra of metal–dielectric enhanced optical resonator and corresponding normal optical resonator.

Figure 7 shows the measured reflection spectra of the metal–dielectric fluorescence enhanced optical resonator and the corresponding normal optical resonator. The peak value of reflectivity, 87%, occurs at the wavelength of 630 nm. The spectrum is in the red-light region, which is in line with the previous theoretical design. In contrast, Figure 7 also demonstrates the spectrum of the normal optical resonator, which has a similar overall structure, except that the InP/ZnSe/ZnS quantum dots in the gain resonator are replaced by the optical filling material SiO_2_ with the same optical distance. The normal optical resonator also reflects red light, with a peak position at 620 nm. The difference from the enhanced optical resonator mainly comes from the small variation in equivalent cavity length caused by the material change. The peak intensity of the reflection spectrum of normal optical resonator is 84%, which is almost equal to the enhanced one, implying that quantum dots perform poorly in conserving light energy. Both of the two devices show very low reflectance in the blue region, under 15%, demonstrating that most of the energy in the blue region is locked in the resonators.

**Figure 8:**
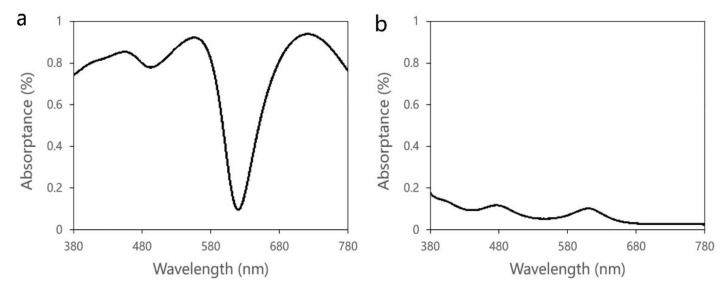
Energy absorptance of (**a**) Cr layer (10 nm) and (**b**) QDPS (388 nm) layer in the metal-dielectric fluorescence enhanced optical resonator.

As to the fact that enhancement coefficient is insufficient, the absorption spectra in different cavity layers were analyzed based on FDTD software, as shown in Figure 8. For the Cr layer at the Mirror 2 side, of which the thickness is 10 nm, the absorptance is as high as 80% overall, except for the red band. Almost all of the light energy is absorbed by this layer, which is originally designed to reflect light and improve the color purity. Comparatively, the quantum dot layer has low absorptance in the whole visible region, which is less than 20%. The high blue light absorption of the metal layer is the fundamental reason for the low enhancement coefficient of the metal-dielectric type.

### 3.3. All-Dielectric Fluorescence Enhanced Optical Resonator

**Figure 9:**
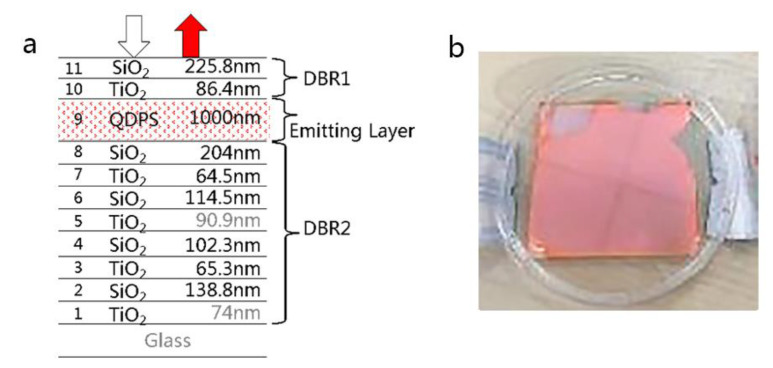
(**a**) Structure of all-dielectric fluorescence enhanced optical resonator and (**b**) the picture of the as-designed sample.

There are two kinds of reflection mirrors that can make up the optical resonator. One is the metal thin film, and the other one is the distributed Bragg reflector (DBR) which is formed by alternating high and low refractive index materials [26]. Compared with the metal thin film, the DBR has many advantages, such as high wavelength selectivity, easy control of reflectivity, and low absorption loss. The structure of an all-dielectric fluorescence enhanced optical resonator device and the sample image are shown in Figure 9. Two DBR structures replace the metal films of the metal–dielectric type as the reflector. Titanium oxide (TiO_2_) works as the high refractive index material, with a refractive index of 2.3, while the low refractive index material is silicon oxide (SiO_2_), with a refractive index of 1.5. QDPS is the InP/ZnSe/ZnS quantum dot fluorescent material. One of the advantages of the all-dielectric fluorescence enhanced optical resonator is that there are no metal materials with strong light absorption in this system, which avoids the competitive absorption between metal materials and quantum dots.

**Figure 10:**
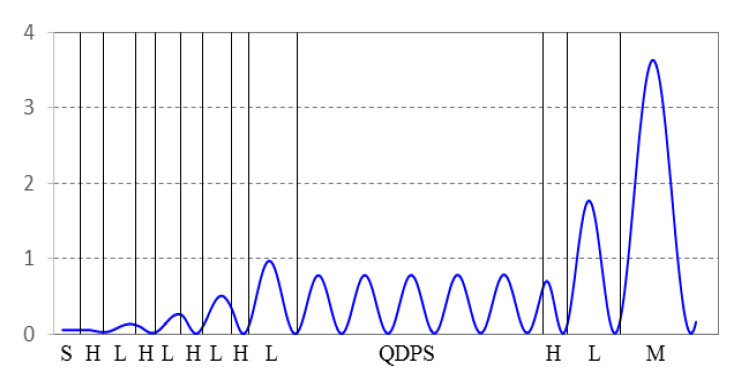
Distribution of electric field (λ = 450 nm) inside all-dielectric fluorescence enhanced optical resonator.

Due to the introduction of the DBR structure, the equivalent cavity length of the whole resonator is greatly increased, compared with the metal–dielectric type. As shown in Figure 10, the distribution of the electric field intensity in the all-dielectric fluorescence enhanced optical resonator is simulated. H and L refer to high and low refractive index materials, respectively. It can be seen that the increase in the cavity length increases the period number of the electric field intensity inside the resonant cavity, which is in the standing wave form. In order to avoid the fluorescent quantum dots all being distributed at the node of the electric field intensity, where the absorption is weakest, the thickness of the QDPS layer is increased to 1 μm. In this case, part of the QDPS layer can always absorb light energy effectively.

**Figure 11:**
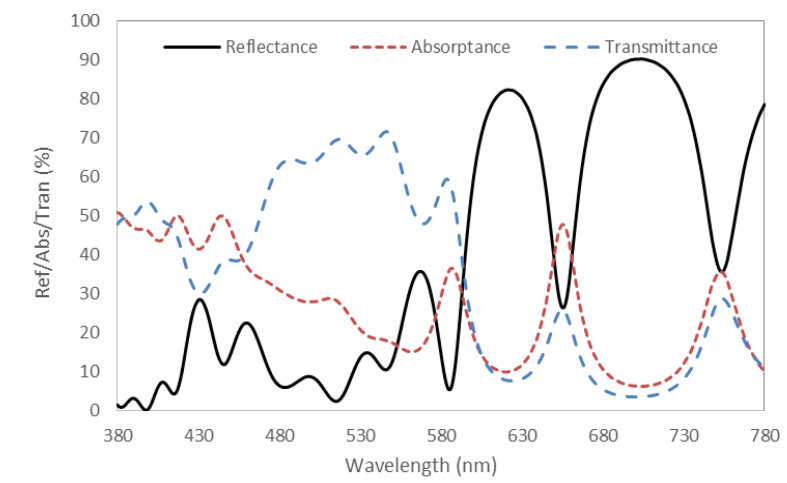
Simulated spectra of all-dielectric normal optical resonator.

Figure 11 shows the simulation results of the all-dielectric normal optical resonator. Due to the large equivalent cavity length of the whole device, the reflection spectrum shows multiple peaks. Through a reasonable design of the reflectivity of DBR, the red-light reflectivity of the device is significantly higher than other bands. In particular, the reflectivity at 625 nm can reach more than 80%. One of the advantages of the DBR structure is that the non-metal design can reduce the absorption by the resonator itself. However, compared with metal-resonator, some light can pass through the device. This is because the lower layer of DBR cannot achieve complete reflection. The difference between the all-dielectric normal resonator and the enhanced one is the choice of the material between the two DBR reflectors. The introduction of the InP/ZnSe/ZnS QDPS layer in the enhanced one makes the optical properties of the device more complex. The total reflection spectrum is the mixture of reflection spectrum determined by the micro-cavity and fluorescent spectrum emitted by QDPS.

**Figure 12:**
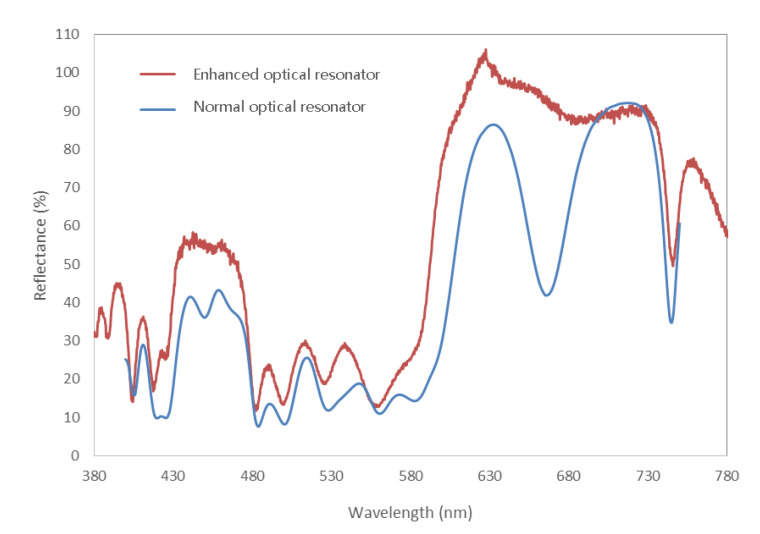
Reflection spectra of all-dielectric fluorescence enhanced optical resonator and corresponding normal optical resonator.

Figure 12 is the experimental reflection spectra of all-dielectric fluorescence enhanced optical resonator and corresponding normal one. The result of all-dielectric normal resonator is basically consistent with the simulation data, demonstrating that the reflection spectrum is mainly concentrated in the red band, even though the increase of the cavity length results in multiple refection peaks. Different from the metal–dielectric type, the all-dielectric enhanced optical resonator shows an obvious change from the corresponding normal resonator. In particular, for the normal resonator, it is the reflection spectral trough stage at 670 nm, while for the corresponding enhanced one, the reflection spectrum does not show a significant decrease. The peak of reflection spectrum of as-designed enhanced type is located at 625 nm, with an equivalent reflectivity up to 105%, which is larger than the incident light. The above phenomena demonstrate that the reflection spectrum of the all-dielectric enhanced resonator superimposes the common effect of the reflected light of the normal type and the fluorescent light wave of the quantum dots. The enhancement coefficient can reach ~124%, calculated by the reflectance value at 625 nm. Interestingly, the reflectance at 450 nm of the enhanced resonator does not decrease, but increases. Even though InP/ZnSe/ZnS quantum dots shows extra fluorescence peak at ~450 nm, it is too weak to cause such a large increase in the reflection. This phenomenon is not well explained. One of the hypotheses is that the electromagnetic field distribution in micro-cavity has an influence on the photoluminescence property of InP/ZnSe/ZnS quantum dots, enhancing the emission at ~450 nm and restraining that at ~625 nm.

## 4. Conclusions

In conclusion, the concept of fluorescence enhanced optical resonator was verified by designing and fabricating a new optical structure coupling quantum dots and thin film resonant cavity. Based on theoretical analysis, the design principles of as-proposed resonator were given. With the intrinsic properties characterized completely, InP/ZnSe/ZnS core-shell quantum dots were utilized as the light conversion material here. Afterwards, two kinds of fluorescence enhanced optical resonator, namely, the metal–dielectric type and all-dielectric type, were manufactured and measured. The metal–dielectric fluorescence enhanced optical resonator showed low improvement of efficiency mainly because of the strong blue light absorbance of metal material. As a contrast, the all-dielectric type, which was realized by using distribute Bragg reflection (DBR), exhibited a peak equivalent reflectance of 105% at 625 nm, with an enhancement coefficient up to 124%. One hypothesis for this phenomenon was proposed. For red fluorescence enhanced optical resonator, when natural light illuminates the structure, red light is reflected while blue and green light are “locked” in the resonator with a specific design. The blue light inside the resonator then is absorbed by red quantum dots, and the energy is converted to red light via stimulated emission. Theoretically, a blue and green fluorescence enhanced optical resonator is also possible. Of note, quantum dots with up-conversion function should be prepared for the blue type because of the utilization of the lower energy part of light. Further research should be done as the next step. In summary, the as-designed fluorescence enhanced optical resonator can combine with a light valve (electrophoresis, electrochromism, etc.) to make a high-efficiency reflective color display, showing comparatively high theoretical and practical value.

## Data Availability

The data presented in this study are available on request from the corresponding author. The data are not publicly available due to privacy issues.

## Supplementary Materials

The following are available online at https://www.mdpi.com/article/10.3390/nano11112813/s1, Figure S1: (a) schematic diagram of thin film resonant cavity; (b) relationship between peak of reflection wavelength and dielectric layer thickness at different interference orders; (c) relationship between wavelength and reflectance at different dielectric layer thickness.
