# Supplementary material for: Fluorescence Enhanced Optical Resonator Constituted of Quantum Dots and Thin Film Resonant Cavity for High-Efficiency Reflective Color Filter
Source: Nanomaterials (Basel). 2021 Oct 23;11(11):2813. doi: 10.3390/nano11112813 (PMC8619847; doi:10.3390/nano11112813)
Supplement: Supplementary file 1 [file nanomaterials-11-02813-s001.zip › nanomaterials-1404400-supplementary.pdf]

Supplementary Materials

Fluorescence Enhanced Optical Resonator Constituted of Quantum Dots and Thin Film Resonant Cavity for High-Efficiency Reflective Color Filter

Xiaochuan Chen [1,2], Pengxia Liang [2], Qian Wu [2], Qiaofeng Tan [1,*] and Xue Dong [2,*]

1 State Key Laboratory of Precision Measurement Technology and Instruments, Department of Precision Instrument, Tsinghua University, Beijing 100084, China; chenxiaochuan@boe.com.cn
2 BOE Technology Group Co., Ltd., Beijing 100176, China; liangpengxia@boe.com.cn (P.L.); wuqian-cto@boe.com.cn (Q.W.)
* Correspondence: tanqf@mail.tsinghua.edu.cn (Q.T.); dongxue@boe.com.cn (X.D.)

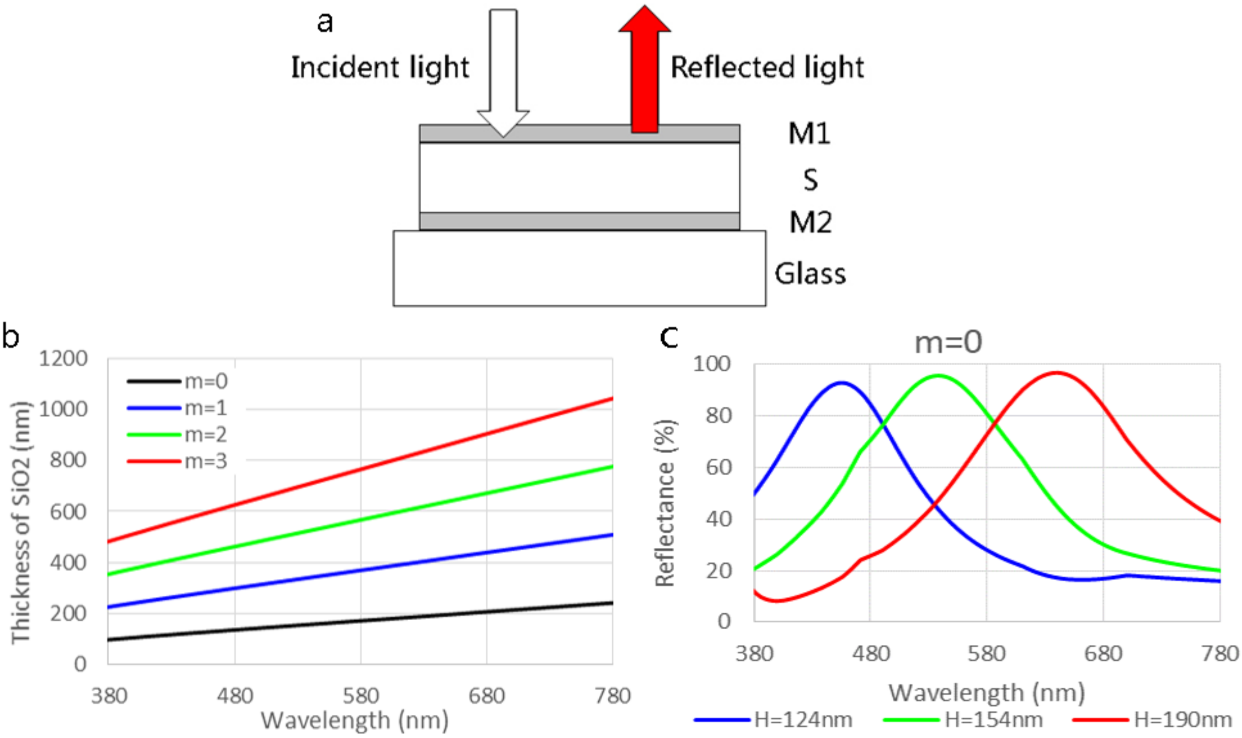

Figure S1. (**a**) schematic diagram of thin film resonant cavity; (**b**) relationship between peak of reflection wavelength and dielectric layer thickness at different interference orders; (**c**)relationship between wavelength and reflectance at different dielectric layer thickness.

The schematic geometry of traditional thin film resonant cavity is shown in Figure S1a. The structure mainly contains a totally reflective mirror, a partially reflective mirror and the dielectric material between them. By adjusting the thickness of dielectric layer, the specific spectrum of reflected light can be achieved. Figure S1b,c shows the relationship between the dielectric layer thickness and wavelength in various cases. Herein, m refers to interference order because light waves are periodic. The performance in different interference orders is similar, showing positive correlation between peak reflection wavelength and dielectric layer thickness. For a specific interference order and a particular peak reflection wavelength, the thickness of dielectric layer is unique. In this case, the reflectance varies greatly at different wavelengths. Based on this characteristic, thin film resonant cavity which could reflect specific spectrum can been manufactured.
